# Supplementary material for: Antioxidant Mechanisms of Echinatin and Licochalcone A
Source: Molecules. 2018 Dec 20;24(1):3. doi: 10.3390/molecules24010003 (PMC6337356; doi:10.3390/molecules24010003)
Supplement: Supplementary file 1 [file molecules-24-00003-s001.zip › supplementary-PDF/Suppl. 1 Phenolics with two phenolic -OHs.pdf]

Suppl. 1 Some phenolics with two phenolic -OHs substitute

Antioxidant Mechanisms of Echinatin and Licochalcone A

Minshi Liang [1,2,†], Xican Li[1,2, †,*], Xiaojian Ouyang [1,2], Hong Xie[1,2], and Dongfeng Chen [3,4,*]

[1] School of Chinese Herbal Medicine; Guangzhou University of Chinese Medicine, Guangzhou 510006, China. E-mails: lminshi@outlook.com (M.L.); oyxiaojian55@163.com (X.O.); xiehongxh1@163.com (H.X.)
[2] Innovative Research & Development Laboratory of TCM; Guangzhou University of Chinese Medicine, Guangzhou 510006, China.
[3] School of Basic Medical Science, Guangzhou University of Chinese Medicine, Guangzhou, China, 510006
[4] The Research Center of Basic Integrative Medicine, Guangzhou University of Chinese Medicine, Guangzhou, China, 510006. E-mail: chen888@gzucm.edu.cn (D. C.)
* Correspondence: lixc@gzucm.edu.cn (X.L.); lixican@126.com (X.L.); chen888@gzucm.edu.cn (D. C.) Tel: +86-20-39358076; Fax: +86-20-38892690
† These authors contributed equally to this work.

No.	Name	Structure	Plant	Type
1	licochalcone C	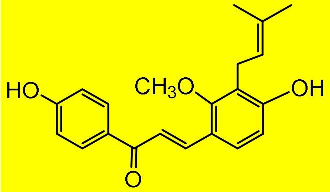	Glycyrrhiza inflata[1]	chalcone
2	2'-O-methylisoliquiritigenin	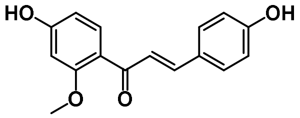	Arachis hypogaea Linn.[2]	chalcone

3	loureirin A	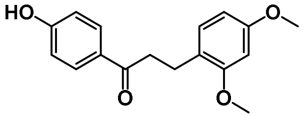	Dracaena cochinchinensis [3]	dihydrochalcone

No.	Name	Structure	Plant	Type
1	corylifol A	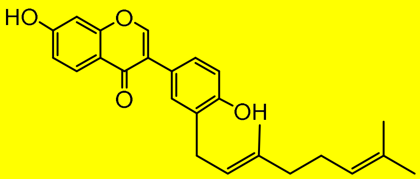	Psoralea corylifolia L.[1]	Isoflavone
2	daidzein	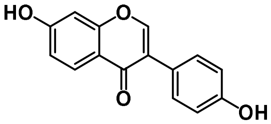	Pueraria lobate [2, 3]	Isoflavone
3	daidzin	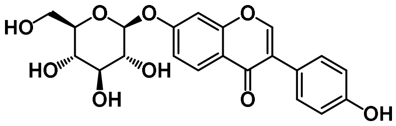	Soybean[3, 4]	Isoflavone
4	7,4'-di-O-methyldaidzein	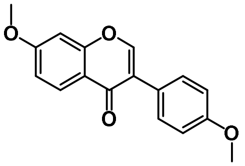	Sophora japonica[5]	Isoflavone

5	8-prenyldaidzein	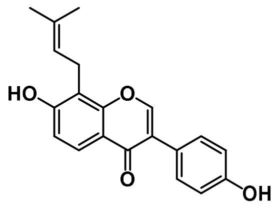	Pueraria lobate [6]	Isoflavone
6	3'-methoxypuerarin	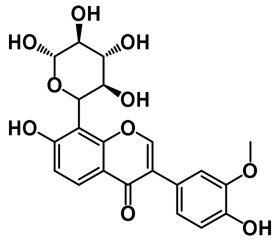	Puerarin lobate (willd).[7]	Isoflavone
7	4'-methoxypuerarin	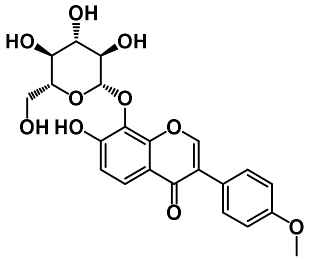	Pueraria lobate [3]	Isoflavone
8	isoformononetin	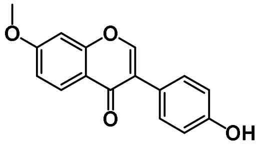	Eysenhardtia polystachya[8]	Isoflavone

References

[1] Xiao, G.; Li, G.; Liang, C.; Zhang, Z.; Yin, J.J.; Tao, W.; Cheng, Z.; Wei, X.; Wang, Z. Isolation of antioxidants from Psoralea corylifolia fruits using high-speed counter-current chromatography guided by thin layer chromatography-antioxidant autographic assay. *Journal of Chromatography A*. **2010**, 1217, 5470-5476.

[2] Pan, W.; Liu, Q. Puerarin and daidzein were isolated from Pueraria lobata by the method of hydrolizing. *Natural Product Research & Development*. **2000**,

[3] Sun, Y.G.; Wang, S.S.; Feng, J.T.; Xue, X.Y.; Liang, X.M. Two new isoflavone glycosides from Pueraria lobata. *Journal of Asian Natural Products Research*. **2008**, 10, 719-723.

[4] Ohta, N.; Kuwata, G.; Akahori, H.; Watanabe, T. Isoflavonoid Constituents of Soybeans and Isolation of a New Acetyl Daidzin. *Journal of the Agricultural Chemical Society of Japan*. **1979**, 43, 1415-1419.

[5] Yu-Ping, T.; Jie, H.; Jing-Hua, W.; Feng-Chang, L. A new coumaronochromone from Sophora japonica. *Journal of Asian Natural Products Research*. **2002**, 4, 1-5.

[6] Hakamatsuka, T.; Ebizuka, Y.; Sankawa, U. Induced isoflavonoids from copper chloride-treated stems of Pueraria lobata. *Phytochemistry*. **1991**, 30, 1481-1482.

[7] Ye, J.; Zhou, X.; Chen, Z.; Ye, W. 2D NMR study on solution structure of natural product 3^-methoxy puerarin. *Journal of Xiamen University*. **2003**, 42,

[8] R., M.P.G.; R., V.S.; S., P.G.; M., Z.S.C.P.G. Antiurolithiatic activity of 7-hydroxy-2',4',5'-trimethoxyisoflavone and 7-hydroxy-4'-methoxyisoflavone from Eysenhardtia Polystachya. *Journal of Herbs Spices & Medicinal Plants*. **2000**, 7, 27-34.
