# Supplementary material for: Antioxidant Mechanisms of Echinatin and Licochalcone A
Source: Molecules. 2018 Dec 20;24(1):3. doi: 10.3390/molecules24010003 (PMC6337356; doi:10.3390/molecules24010003)
Supplement: Supplementary file 1 [file molecules-24-00003-s001.zip › supplementary-PDF/Suppl. 2 Phenolics with 1,1-dimethyl-2-propenyl substitute.pdf]

Suppl. 2 Phenolics with 1,1-dimethyl-2-propeny substitute

Antioxidant Mechanisms of Echinatin and Licochalcone A

Minshi Liang [1,2,†], Xican Li[1,2, †,*], Xiaojian Ouyang [1,2], Hong Xie[1,2], and Dongfeng Chen [3,4,*]

[1] School of Chinese Herbal Medicine; Guangzhou University of Chinese Medicine, Guangzhou 510006, China. E-mails: lminshi@outlook.com (M.L.); oyxiaojian55@163.com (X.O.); xiehongxh1@163.com (H.X.)
[2] Innovative Research & Development Laboratory of TCM; Guangzhou University of Chinese Medicine, Guangzhou 510006, China.
[3] School of Basic Medical Science, Guangzhou University of Chinese Medicine, Guangzhou, China, 510006
[4] The Research Center of Basic Integrative Medicine, Guangzhou University of Chinese Medicine, Guangzhou, China, 510006. E-mail: chen888@gzucm.edu.cn (D. C.)
* Correspondence: lixc@gzucm.edu.cn (X.L.); lixican@126.com (X.L.); chen888@gzucm.edu.cn (D. C.) Tel: +86-20-39358076; Fax: +86-20-38892690
† These authors contributed equally to this work.

No.	Name	Structure	Plant	Type
1	Isocudraniazanthone A	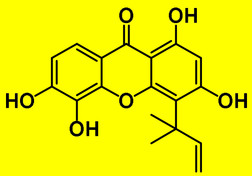	Garcinia mangostana Linn[1]	xanthone
2	5,9,11-trihydroxy-10-(2''-hydroxy-3''-methylbut-3''-en-1-yl)-2,2-dimethyl-3-(2'-methylbut-3'-en-2'-yl)-2H,12H-pyrano[2,3-a]xanthen-12-one	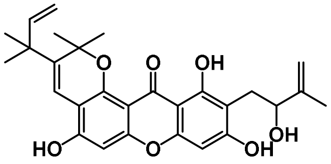	The stem bark of Calophyllum pseudomole[2]	xanthone

3	allanxanthone A	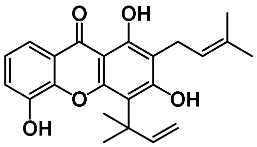	the stem bark of Allanblackia floribunda[3]	xanthone
4	4-(1,1-dimethylprop-2-enyl)-1,3,5,8-tetrahydroxyxanthone	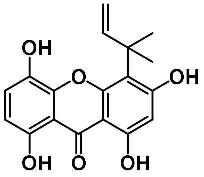	Garcinia cantleyana[4, 5]	xanthone
5	1,3,5 - trihydroxy - 6,6’ - dimethyl-pyrano(2’,3’:6,7) - 4- (1,1-dimethyl-pro-2-enyl)xanthone	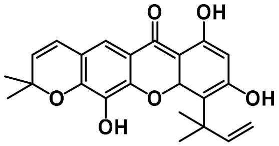	C. inophyllum, G.opaca and G. bancana[6, 7]	xanthone
6	macluraxanthone	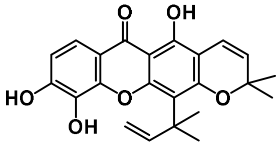	C. inophyllum, G.opaca and G. bancana[6, 7]	xanthone
7	1,3,6,7-tetrahydroxy-2-(3-methylbut-2-enyl)-8-(2-methylbut-3-en-2-yl)-9H-xanthen-9-one	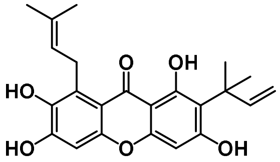	Cundrania tricuspidata[8]	xanthone

8	Macluraxanthone B	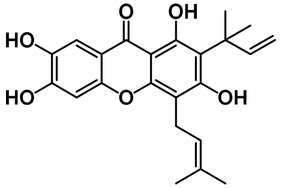	Cundrania tricuspidata[8, 9]	xanthone
9	Cudraxanthone L	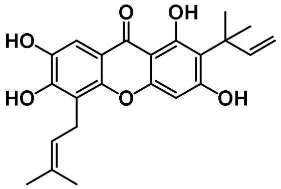	Cundrania tricuspidata[8, 9]	xanthone
10	1,3,7-trihydroxy-4-(1,1-dimethyl-2-propenyl)-5,6-(2-2-dimethylchromeno) xanthone	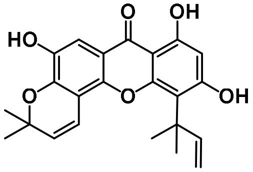	Cundrania tricuspidata[8]	xanthone
11	Cudratricusxanthone F	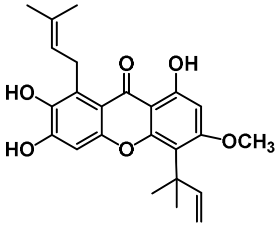	Cundrania tricuspidata[8]	xanthone

12	Cudratricusxanthone A	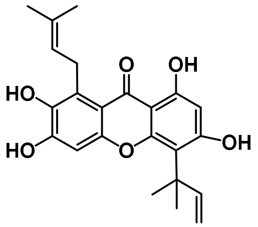	Cundrania tricuspidata[8]	xanthone
13	2,3,6,8-tetrahydroxy-1-(3-methylbut-2-enyl)-5-(2-methylbut-3-en-2-yl)-9H-xanthen-9-one	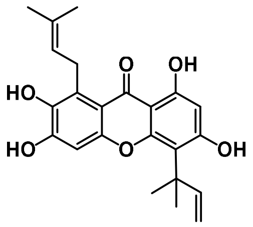	Cudrania tricuspidata[9]	xanthone
14	penangianaxanthone	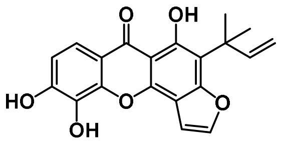	Garcinia penangiana Pierre[5]	xanthone
15	cudratricusxanthone H	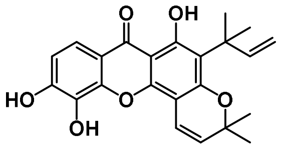	Garcinia penangiana Pierre[5]	xanthone
16	macluraxanthone C	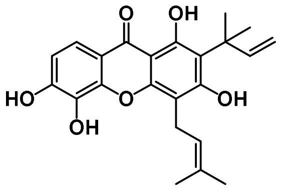	Garcinia penangiana Pierre[5]	xanthone

17	banganxanthone C	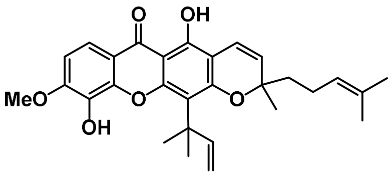	the Leaves of Garcinia polyantha[10]	xanthone
18	1,2-dihydro-3,6,8-trihydroxy-1,1-bis (3-methylbut -2-enyl)-5-(1, 1-dimethylprop-2-enyl)–xanthen-2,9-dione	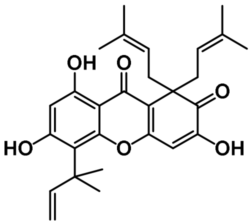	Hypericum Erectum[11]	xanthone
19	6,8-dihydroxy-2,7-dimethoxy-3-(1,1-dimethylprop-2-enyl)-1,4-naphthoquinone	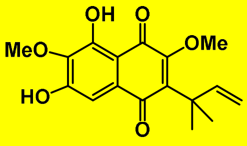	Lysionotus pauciflorus[12]	naphthoquinone
20	2-(1,1-dimethylprop-2-enyl)-3-hydroxy-1,4 naphthoquinone	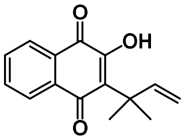	Calceolaria andina L.[13]	
21	5,7,4'-trihydroxy-8-(1,1-dimethylprop-2-enyl) isoflavone	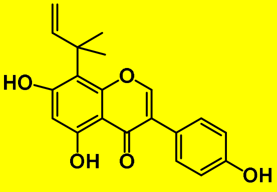	Flemingia paniculata[14]	Isoflavone

22	5,7,2',4'-tetrahydroxy-8-(1,1-dimethylprop-2-enyl)-isoflavone	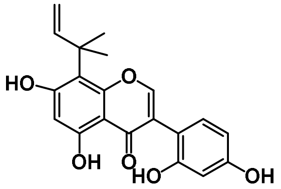	Flemingia paniculata[14]	
23	glycyuralin B	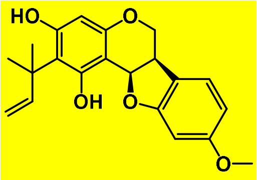	the roots and rhizomes of Glycyrrhiza uralensis (licorice)[15]	isoflavane

[1] Martinez, A.; Hernandez-Marin, E.; Galano, A. Xanthones as antioxidants: a theoretical study on the thermodynamics and kinetics of the single electron transfer mechanism. *Food Funct.* **2012**, 3, 442-450.

[2] Tanjung, M.; Saputri, R.D.; Tjahjandarie, T.S. 5,9,11-Trihydroxy-10-(2″-hydroxy-3″-methylbut-3″-en-1-yl)-2,2-dimethyl-3-(2′-methylbut-3′-en-2′-yl)-2H,12H-pyrano[2,3-a]xanthen-12-one from Calophyllum pseudomole. *Molbank.* **2017**, 2017, M961.

[3] Nkengfack, A.E.; Azebaze, G.A.; Vardamides, J.C.; Fomum, Z.T.; van Heerden, F.R. A prenylated xanthone from Allanblackia floribunda. *Phytochemistry.* **2002**, 60, 381-384.

[4] Shadid, K.A.; Shaari, K.; Abas, F.; Israf, D.A.; Hamzah, A.S.; Syakroni, N.; Saha, K.; Lajis, N.H. Cytotoxic caged-polyprenylated xanthonoids and a xanthone from Garcinia cantleyana. *Phytochemistry.* **2007**, 68, 2537-2544.

[5] Jabit, M.L.; Khalid, R.; Abas, F.; Shaari, K.; Hui, L.S.; Stanslas, J.; Lajis, N.H. Cytotoxic xanthones from Garcinia penangiana Pierre. *Z Naturforsch C.* **2007**, 62, 786-792.

[6] Jantan, I.; Juriyati, J.; Warif, N.A. Inhibitory effects of xanthones on platelet activating factor receptor binding in vitro. *J Ethnopharmacol.* **2001**, 75, 287-290.

[7] Jantan, I.B.; Jalil, J.; Warif, N.M.A. Platelet Activating Factor (PAF) Antagonistic Activities of Compounds Isolated from Guttiferae Species. *Pharmaceutical Biology.* **2001**, 39, 243-246.

[8] Seo, E.J.; Curtis-Long, M.J.; Lee, B.W.; Kim, H.Y.; Ryu, Y.B.; Jeong, T.S.; Lee, W.S.; Park, K.H. Xanthones from Cudrania tricuspidata displaying potent alpha-

glucosidase inhibition. *Bioorg Med Chem Lett*. **2007**, 17, 6421-6424.
[9] Lee, B.W.; Gal, S.W.; Park, K.M.; Park, K.H. Cytotoxic xanthones from Cudrania tricuspidata. *J Nat Prod*. **2005**, 68, 456-458.
[10] Lannang, A.M.; Tatsimo, S.J.; Fouotsa, H.; Dzoyem, J.P.; Saxena, A.K.; Sewald, N. Cytotoxic compounds from the leaves of Garcinia polyantha. *Chem Biodivers*. **2014**, 11, 975-981.
[11] Tian Ying, A.N.; Li Hong, H.U. A New Xanthone Derivative from Hypericum Erectum. *Chinese Chemical Letters*. **2002**, 13, 623-624.
[12] Zhong, Y.J.; Wen, Q.F.; Li, C.Y.; Su, X.H.; Yuan, Z.P.; Li, Y.F. Two New Naphthoquinone Derivatives from Lysionotus pauciflorus. *Helvetica Chimica Acta*. **2013**, 96, 1750-1756.
[13] Khambay, B.P.; Batty, D., .; Cahill, M., .; Denholm, I., .; Mead-Briggs, M., .; Vinall, S., .; Niemeyer, H.M.; Simmonds, M.S. Isolation, characterization, and biological activity of naphthoquinones from Calceolaria andina L. *Journal of Agricultural & Food Chemistry*. **1999**, 47, 770.
[14] Rahman, M.M.; Sarker, S.D.; Byres, M.; Gray, A.I. New salicylic acid and isoflavone derivatives from Flemingia paniculata. *J Nat Prod*. **2004**, 67, 402-406.
[15] Ji, S.; Li, Z.; Song, W.; Wang, Y.; Liang, W.; Li, K.; Tang, S.; Wang, Q.; Qiao, X.; Zhou, D. Bioactive Constituents of Glycyrrhiza uralensis (Licorice): Discovery of the Effective Components of a Traditional Herbal Medicine. *Journal of Natural Products*. **2017**, 79, 281.
