# Supplementary material for: Antioxidant Mechanisms of Echinatin and Licochalcone A
Source: Molecules. 2018 Dec 20;24(1):3. doi: 10.3390/molecules24010003 (PMC6337356; doi:10.3390/molecules24010003)
Supplement: Supplementary file 1 [file molecules-24-00003-s001.zip › supplementary-PDF/Suppl. 3 Dose response curves Figure S1-S5.pdf]

Suppl. 3 Dose–response curves

Antioxidant Mechanisms of Echinatin and Licochalcone A

Minshi Liang [1,2,†], Xican Li[1,2, †,*], Xiaojian Ouyang [1,2], Hong Xie[1,2], and Dongfeng Chen [3,4,*]

[1] School of Chinese Herbal Medicine; Guangzhou University of Chinese Medicine, Guangzhou 510006, China. E-mails: lminshi@outlook.com (M.L.); oyxiaojian55@163.com (X.O.); xiehongxh1@163.com (H.X.)

[2] Innovative Research & Development Laboratory of TCM; Guangzhou University of Chinese Medicine, Guangzhou 510006, China.

[3] School of Basic Medical Science, Guangzhou University of Chinese Medicine, Guangzhou, China, 510006

[4] The Research Center of Basic Integrative Medicine, Guangzhou University of Chinese Medicine, Guangzhou, China, 510006. E-mail: chen888@gzucm.edu.cn (D. C.)

* Correspondence: lixc@gzucm.edu.cn (X.L.); lixican@126.com (X.L.); chen888@gzucm.edu.cn (D. C.) Tel: +86-20-39358076; Fax: +86-20-38892690

† These authors contributed equally to this work.

Note: This Supporting information provides the original data of **Table 1** in the main text. All data underline are mentioned in Table 1 in the main text.

1. *Fe^{3+}-reducing power assay*

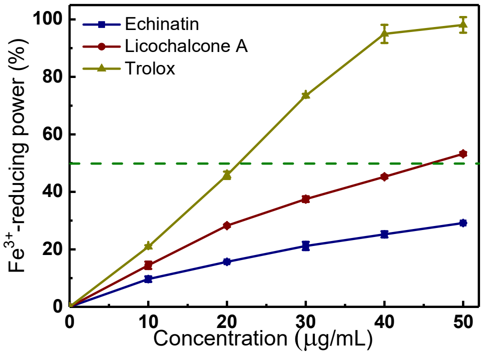

Figure S1: The dose response curves of echinatin and licochalcone A in Fe^{3+}-reducing power assay. Each value is expressed as mean ± SD (n = 3).

Tab. S1 The comparison of IC_{50} values of echinatin and licochalcone A and positive control in Fe^{3+}-reducing power assay.

	Mean±SD µg/mL	Mean±SD µM
echinatin	91.3±2.3	338.0±8.6 ^{c}
licochalcone A	45.1±0.3	133.2±0.9 ^{b}
Trolox	21.9±0.9	87.5±3.4 ^{a}

IC_{50} value was defined as the concentration of 50% superoxide anion radical inhibition and calculated by linear regression which was analyzed by Origin 2017 professional software. Means values with different superscripts in the same column are significantly different ($p<0.05$).

2. *Cu^{2+}-reducing power assay*

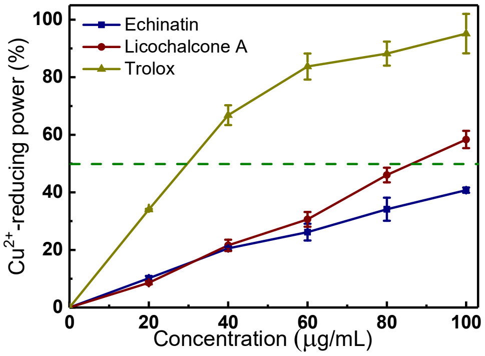

Figure S2: The dose response curves of echinatin and licochalcone A in Cu^{2+}-reducing power assay. Each value is expressed as mean ± SD (n = 3).

Tab. S2 The comparison of IC_{50} values of echinatin and licochalcone A and positive control in Cu^{2+}-reducing power assay.

	Mean±SD µg/mL	Mean±SD µM
echinatin	66.0±2.9	228.1±10.6 ^{c}
licochalcone A	43.7±0.7	129.1±2.1 ^{b}
Trolox	16.9±0.2	67.5±0.9 ^{a}

IC_{50} value was defined as the concentration of 50% superoxide anion radical inhibition and calculated by linear regression which was analyzed by Origin 2017 professional software. Means values with different superscripts in the same column are significantly different ($p<0.05$).

3. *PTIO•-scavenging assay*

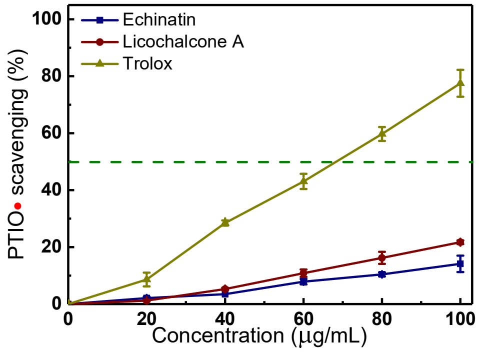

Figure S3: The dose response curves of echinatin and licochalcone A in PTIO•-scavenging assay. Each value is expressed as mean ± SD (n = 3).

Tab. S3 The comparison of IC_{50} values of echinatin and licochalcone A and positive control in PTIO•-scavenging assay.

	Mean±SD μg/mL	Mean±SD μM
echinatin	345.0±40.5	1276.5±149.9 [c]
licochalcone A	208.9±7.8	617.4±22.9 [b]
Trolox	67.8±2.7	270.9±10.8 [a]

IC_{50} value was defined as the concentration of 50% superoxide anion radical inhibition and calculated by linear regression which was analyzed by Origin 2017 professional software. Means values with different superscripts in the same column are significantly different ($p<0.05$).

4. *DPPH•-scavenging assay*

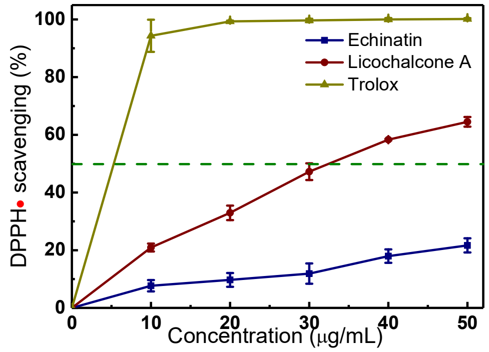

Figure S4: The dose response curves of echinatin and licochalcone A in DPPH•-scavenging assay. Each value is expressed as mean ± SD (n = 3).

Tab. S4 The comparison of IC_{50} values of echinatin and licochalcone A and positive control in DPPH•-scavenging assay.

	Mean±SD μg/mL	Mean±SD μM
echinatin	106.6±18.2	394.2±67.5 [c]
licochalcone A	34.6±1.2	102.3±3.6 [b]
Trolox	12.0±0.6	47.8±2.6 [a]

IC_{50} value was defined as the concentration of 50% superoxide anion radical inhibition and calculated by linear regression which was analyzed by Origin 2017 professional software. Means values with different superscripts in the same column are significantly different ($p<0.05$).
