# Supplementary material for: Antioxidant Mechanisms of Echinatin and Licochalcone A
Source: Molecules. 2018 Dec 20;24(1):3. doi: 10.3390/molecules24010003 (PMC6337356; doi:10.3390/molecules24010003)
Supplement: Supplementary file 1 [file molecules-24-00003-s001.zip › supplementary-PDF/Suppl. 4 Certificate analysis of echinatin.pdf]

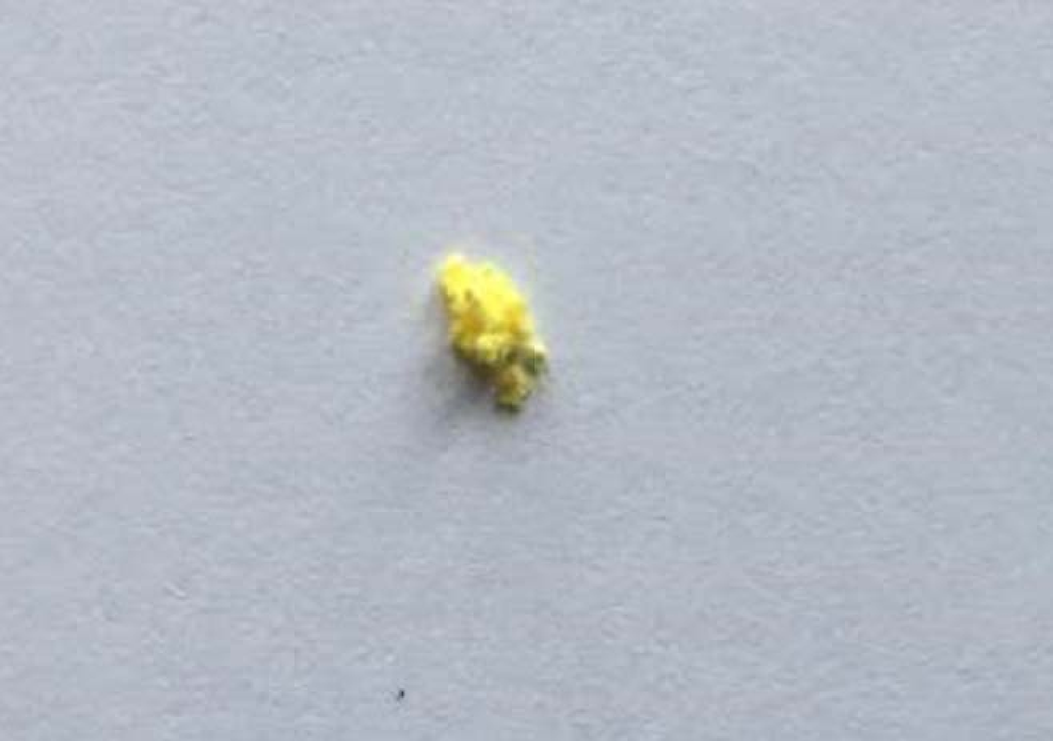

ALFA

www.alfabiotech.com

成都埃法生物科技有限公司

Chengdu Alfa Biotechnology Co.,Ltd.

产品分析证书

Certificate of Analysis

产品信息

中文名称	刺甘草查尔酮	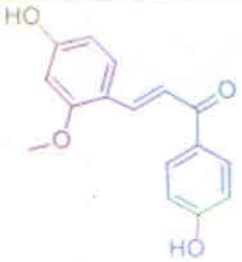
英文名称	Echinatin	
CAS 号码	34221-41-5	
分子式	C16H14O4	
分子量	270.284	
批号	AF8041723	

分析结果

检测项目	质量标准	结果
性状	黄色粉末。	符合。
液相色谱	≥98.0%（面积归一化法）。	99.70%。
质谱	应符合其结构。	符合。
核磁	应符合其结构。	符合。
结论	产品质量符合报告标准，准予出厂。	

注意事项

贮存方法	密闭、干燥、置于 2-8℃避光保存。
使用方法	即配即用，仅限用于实验室使用，不得用于人体。
提示	如遇质量问题，请于收到产品之日起 15 日内与我们联系，感谢你的选购。

核准：王 斌

质检专用章

日期：2018-07-20

Tel:86-28-85142057 Email:sales@alfabiotech.com
Add: No.230, Longyin Road,Pidu District,Chengdu ,China,610000

HPLC 图谱

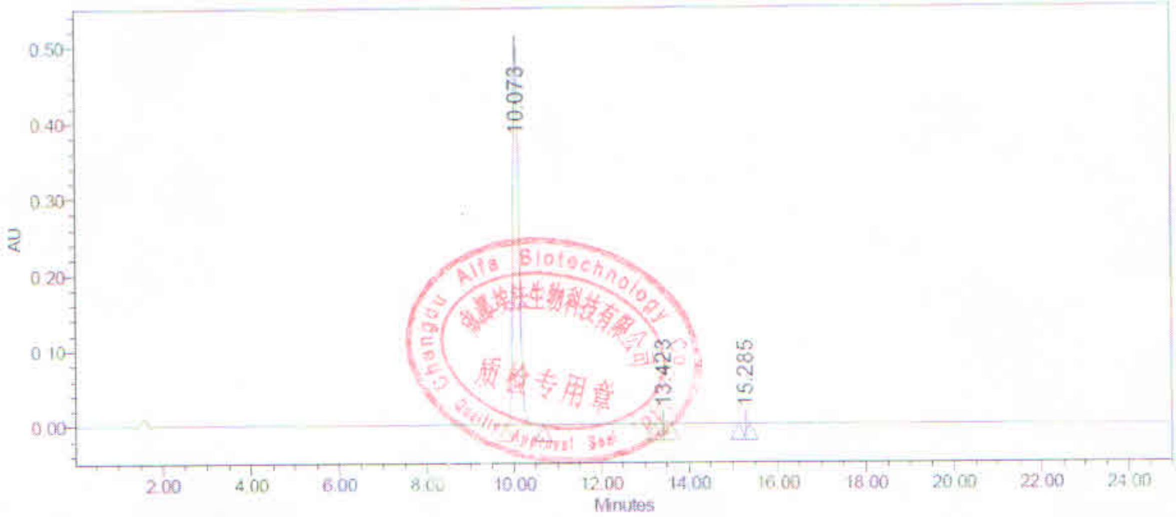

Peak Results

	Name	RT	Area	% Area	USP Plate Count	USP Resolution
1		10.073	4715662	99.70	26184.98	
2		13.423	11953	0.25	42793.92	12.78
3		15.285	2211	0.05	82963.44	7.52

Tel:86-28-85142057 Email:sales@alfabiotech.com
Add: No.230, Longyin Road,Pidu District,Chengdu ,China,610000
