# Supplementary material for: Antioxidant Mechanisms of Echinatin and Licochalcone A
Source: Molecules. 2018 Dec 20;24(1):3. doi: 10.3390/molecules24010003 (PMC6337356; doi:10.3390/molecules24010003)
Supplement: Supplementary file 1 [file molecules-24-00003-s001.zip › supplementary-PDF/Suppl. 5 Certificate analysis of licochalcone A.pdf]

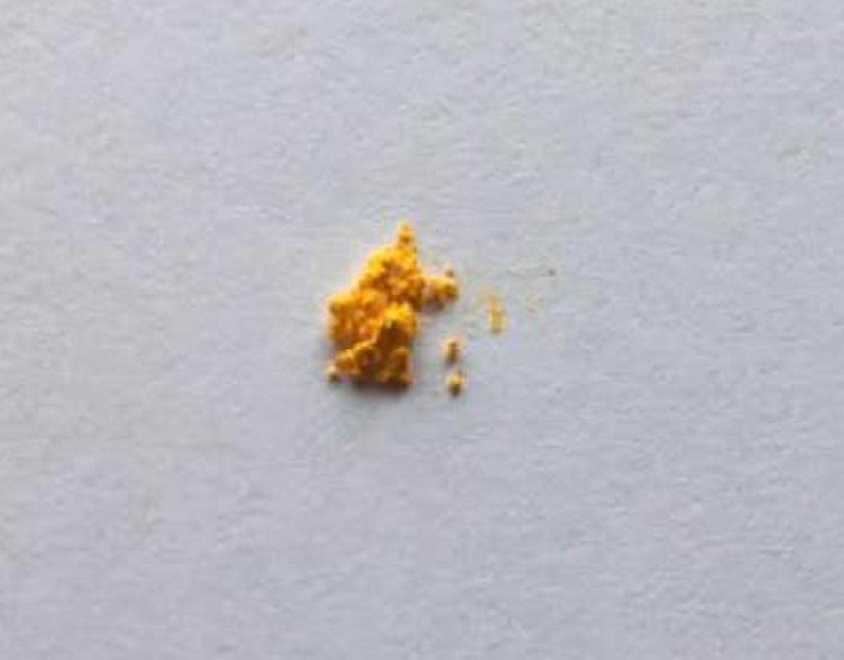

西力生物
BBP BioBioPha

Natural Product Library for Drug Discovery

CERTIFICATE OF ANALYSIS

BBP No.: BBP03989

CAS No.: 58749-22-7

Chemical Name: Licochalcone A

Molecular Formula: $C_{21}H_{22}O_4$

Structure:

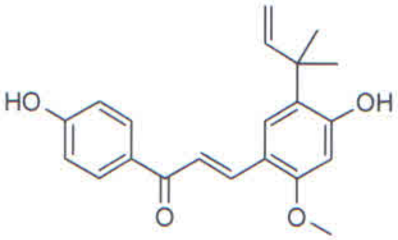

Purity: 98%

Appearance: Yellow powder

Solvent: Acetone, methanol

Exact Weight: 5.1 mg

Storage: Store in a dark place under the temperature of 0−4 °C

Intended Use: For laboratory use only

Reference: G. Yoon, Arch Pharm Res, 2007, 30(3), 313-316

Warm Notice: When publishing, please cite as: ***chemical name*** was purchased from BioBioPha Co., Ltd. (Kunming, China)

Characterization Data Summary

Analytical Test	Results
Identification by ^{1}H-NMR	Consistent with the above structure
Purity tested by HPLC, ^{1}H-NMR	98%

Authorized Signature:

Date:

BioBioPha Co., Ltd.
2018.07.20
PASS

Tel.: +86 871 6521 7109 Fax: +86 871 6521 5563 E-mail: sales@mail.biobiopha.com

西力生物
BBP BioBioPha

Natural Product Library for Drug Discovery

PRODUCT QUALITY REPORT

Product Number: BBP03989

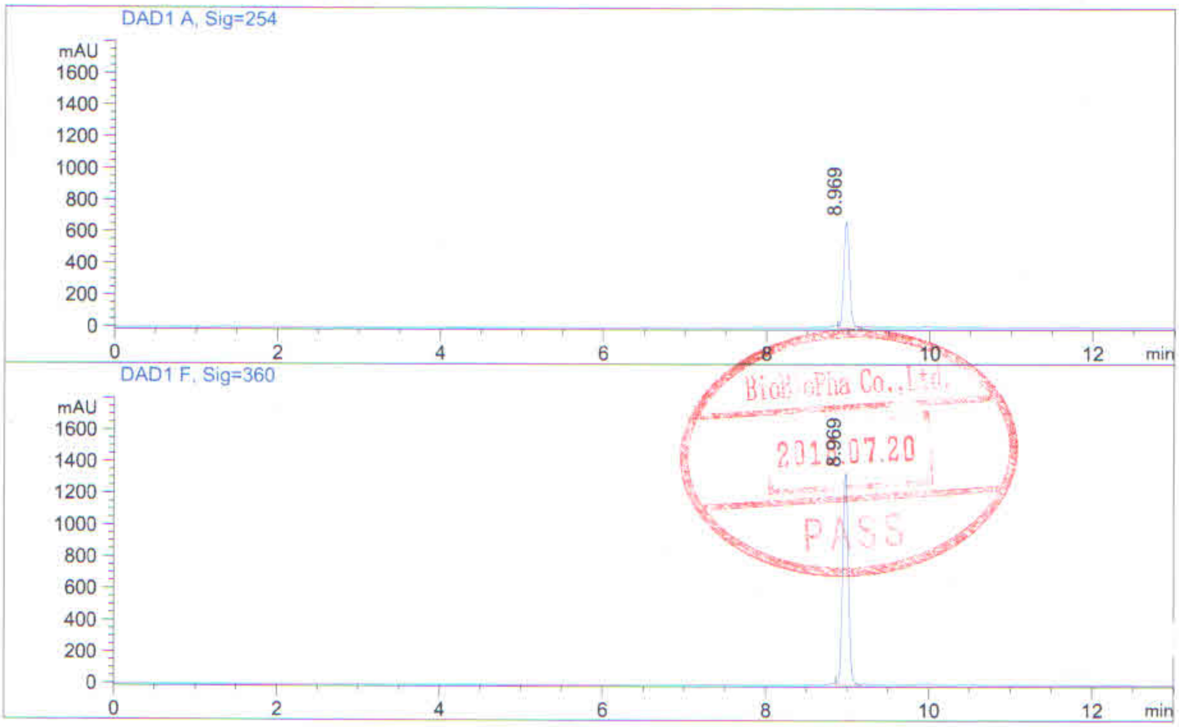

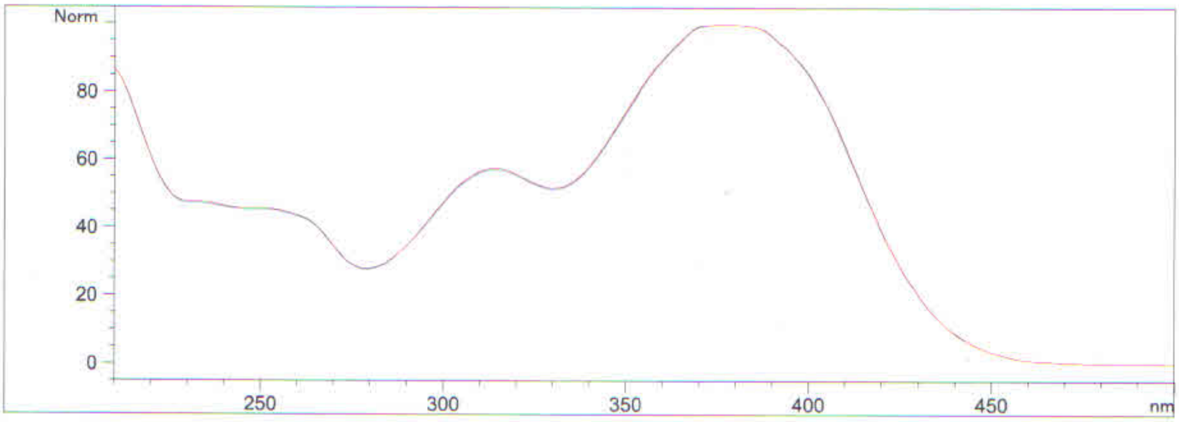

Agilent 1200 series HPLC system
Extend-C18 column (5 μm, 4.6 × 150 mm)
20%→100% MeOH in H_2O over 8.0 min followed by 100% MeOH to 13.0 min
1.0 ml/min, 20°C

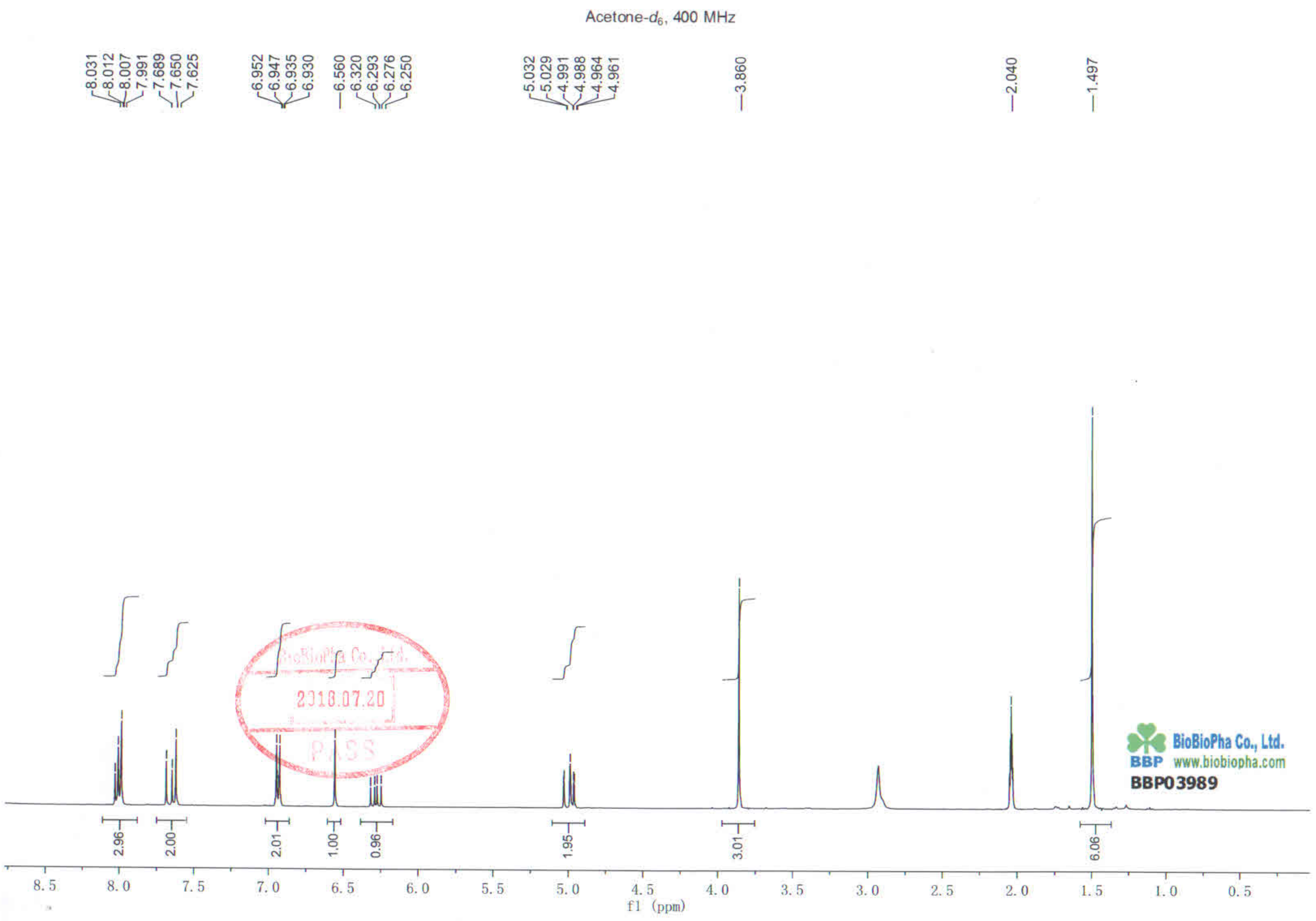

Acetone-d_6, 400 MHz
8.031
8.012
8.007
7.991
7.689
7.650
7.625
6.952
6.947
6.935
6.930
6.560
6.320
6.293
6.276
6.250
5.032
5.029
4.991
4.988
4.964
4.961
3.860
2.040
1.497
2.96
2.00
2.01
1.00
0.96
1.95
3.01
6.06
8.5
8.0
7.5
7.0
6.5
6.0
5.5
5.0
4.5
4.0
3.5
3.0
2.5
2.0
1.5
1.0
0.5
f1 (ppm)
BioBioPha Co., Ltd.
2018.07.20
PASS
BioBioPha Co., Ltd.
BBP www.biobiopha.com
BBP03989
